# Application of Blood-Based Biomarkers in Human Mild Traumatic Brain Injury

**DOI:** 10.3389/fneur.2013.00044

**Published:** 2013-05-01

**Authors:** Alex P. Di Battista, Shawn G. Rhind, Andrew J. Baker

**Affiliations:** ^1^Faculty of Medicine, Institute of Medical Science, University of TorontoToronto, ON, Canada; ^2^Physiology Group, Individual Behaviour and Performance Section, Defence Research and Development Canada TorontoToronto, ON, Canada; ^3^Department of Anesthesia, University of TorontoToronto, ON, Canada; ^4^Department of Surgery, University of TorontoToronto, ON, Canada; ^5^Keenan Research Centre, Li Ka Shing Knowledge Institute, St. Michael’s HospitalToronto, ON, Canada

**Keywords:** peripheral blood, diagnostic markers, prognostic markers, pathophysiology, biological mechanisms, high throughput, post-concussion syndrome, chronic traumatic encephalopathy

## Abstract

Traumatic Brain Injury (TBI) is a global health concern. The majority of TBI’s are mild, yet our ability to diagnose and treat mild traumatic brain injury (mTBI) is lacking. This deficiency results from a variety of issues including the difficulty in interpreting ambiguous clinically presented symptoms, and ineffective imaging techniques. Thus, researchers have begun to explore cellular and molecular based approaches to improve both diagnosis and prognosis. This has been met with a variety of challenges, including difficulty in relating biological markers to current clinical symptoms, and overcoming our lack of fundamental understanding of the pathophysiology of mTBI. However, recent adoption of high throughput technologies and a change in focus from the identification of single to multiple markers has given just optimism to mTBI research. The purpose of this review is to highlight a number of current experimental peripheral blood biomarkers of mTBI, as well as comment on the issues surrounding their clinical application and utility.

## Introduction

Traumatic brain injury (TBI) affects up to 10 million people globally (Hyder et al., 2007). Mild traumatic brain injury (mTBI) accounts for between 70 and 90% of all TBI cases, and has an estimated incidence rate of 653/100,000 people in Ontario alone (Ryu et al., 2009). mTBI has gained considerable notoriety during the past decade of conflict in Afghanistan and Iraq as a common source of morbidity from these wars. An estimated 10–20% of all veterans of these conflicts sustain mTBI, with blast injuries being the leading cause (Schneiderman et al., 2008; Kelly et al., 2012; Shively and Perl, 2012). Yet, mTBI is proportionally understudied compared to moderate and severe injury (Papa et al., 2008).

The underlying pathophysiology of mTBI remains undetermined, and as a result there are currently no efficient diagnostic, prognostic, or therapeutic strategies available clinically. Researchers have begun to investigate mTBI at the cellular and molecular level, as shortcomings in current brain imaging techniques and flawed clinical diagnostic approaches have increased the appeal of utilizing the peripheral blood to identify immune and damage related signaling between the brain and the periphery. Specifically, the goal of this approach is to uncover either a single marker or panel of markers to aid in early detection and diagnosis, as well as to predict patient outcomes. Furthermore, this method may help elucidate underlying biological mechanisms and provide greater insight into therapeutic strategies. However, the utility of such markers needs to be determined in the experimental phase through careful evaluation against specific clinical questions, including: whether/how they directly relate to disease mechanisms, prognosis, diagnosis, and the monitoring of therapeutic inventions. This validation will facilitate a positive transition within the “bench to bedside” process.

The determinants and modifiers of the clinical entity of concussion, mTBI, and post-concussion syndrome (PCS) include several factors such as environmental, psychosocial, and co-morbidities among others. These factors may be operative before and/or after trauma. The scope of this review is narrowly focused on the biological factors of presumed loss of cell integrity that can be detected using signature biochemical biomarker patterns in human peripheral blood, in terms of their specific utility and clinical relevance in relating to pathophysiology, diagnosis, and prognosis. Biomarker categorization will be based on a stratification originally put forth by the Biomarkers Definition Working Group (BDWG) (Biomarkers Definitions Working Group, 2001), and serve to aid in the guided development of translating important laboratory findings into meaningful clinical practice.

## mTBI Definition and Terminology

In order to assess and treat mTBI clinically, a clear definition is necessary. In view of this, clinicians and researchers have historically struggled to agree on a singular mTBI definition and accepted terminology (Moser et al., 2007; Ruff et al., 2009; Marshall et al., 2012). This disagreement may stem from a variety of issues including the heterogeneity in both trauma mechanism and symptom presentation, and the difficulty in detecting signs and symptoms of injury (Greenwald et al., 2012). In addition, there are disputes over the use of the term mTBI as opposed to concussion; it is unclear if these terms can be used interchangeably, or if there is a difference in the underlying definition (Greenwald et al., 2012; Jeter et al., 2012). It has been suggested that an mTBI that occurs in sports is typically referred to as a concussion (Moser et al., 2007). In an attempt at clarity and unification, a definition has been put forth by the American Congress of Rehabilitation Medicine (ACRM) that is endorsed by both the Centre of Disease Control and Prevention (CDC) and the World Health Organization (WHO). In summary, the ACRM defines an mTBI as:

“A traumatically induced physiological disruption of brain function, as manifested by at least one of the following…loss of consciousness…any loss of memory for events immediately before or after the event…focal neurologic deficit that may or may not be transient, but where the severity of the injury does not exceed the following: loss of consciousness of approximately 30 min or less after 30 min, an initial Glasgow Coma Scale score of 13–15 and post-traumatic amnesia not greater than 24 h” (Greenwald et al., 2012).

## Peripheral Blood as a Source for mTBI Biomarkers

Using peripheral blood for mTBI biomarker discovery potentially addresses a number of clinical concerns, such as current diagnostic, prognostic, and treatment approach pitfalls, the difficulty in creating representative animal models of human mTBI, and the inability to conduct biological studies on patients at the primary site of injury. For example, diagnosis and prognosis in moderate and severe TBI using conventional imaging techniques is informative (Shenton et al., 2012), but these techniques fail to detect the majority of mild brain injuries and provide little or no pathophysiological information related to injury mechanism (Papa et al., 2008; Bettermann and Slocomb, 2012). Furthermore, animal models of human mTBI, which have largely focused on the use of rodents, have been met with little success. This is due to a number of factors, such as the heterogeneity of the human clinical population from which it mimics, and the anatomical differences between the human and rodent brain (Marklund and Hillered, 2011). Lastly, the use of a biological correlate at or near the site of injury, such as the Cerebrospinal Fluid (CSF) is potentially advantageous due to its proximity to the brain and involvement in the central nervous system (CNS). However, the acquisition of CSF fluid is a relatively invasive procedure. By comparison, acquiring a blood sample from a patient population is more accepted in clinical practice, and can provide substantial information relating to specific neurological injury processes within the brain, the blood brain barrier (BBB), and neuroendocrine-immune signaling processes between the CNS and periphery.

## Biomarkers of mTBI Diagnosis

Ideally, a diagnostic biomarker should indicate the presence or absence of disease/injury, and more specifically, should be able to stage or classify its severity (Biomarkers Definitions Working Group, 2001). No clinically accepted TBI peripheral blood biomarkers currently exist (Bettermann and Slocomb, 2012). The spectrum of TBI is presently diagnosed and stratified through the Glasgow Coma Scale (GCS) rating system alongside presentation, neurological examination, and CT imaging (Sharma and Laskowitz, 2012). While the GCS can be effective in assessing neurocognitive state, and offers some prognostic information regarding patient outcome, it tells us little about the physiological source of these symptoms, and can be confounded by polytrauma, alcohol, and other drug use (Papa, 2012). In addition, clinical CT imaging often fails to detect moderate and mild TBI, causes potential exposure to harmful radiation, and is a relatively costly procedure (Bettermann and Slocomb, 2012).

Diagnosing mTBI through a blood-based test may circumvent the pitfalls of the current clinical diagnostic approach, potentially offering a specific and sensitive evaluation of presented neurological deficit that is based on objective, quantifiable biological changes directly related to trauma physiology (Topolovec-Vranic et al., 2011). However, this approach is confounded by a variety of factors, including a poor understanding of the underlying biological mechanisms of mTBI, as well as the difficulty in linking blood-based protein markers to a range of dynamic clinical symptoms that are difficult to objectively assess. Moreover, in specific populations such as military personnel, the relationship between combat-related mTBI and residual mTBI symptoms, post-traumatic stress disorder (PTSD) symptoms, and neurocognitive deficits remains unclear (Brenner, 2011; Miller, 2011). TBI occurrence and severity are difficult to ascertain in this population because of retrospective bias in determining relevant clinical variables, such as whether there was loss of consciousness or post-traumatic amnesia (Schneiderman et al., 2008; Kelly et al., 2012; Shively and Perl, 2012).

Numerous markers have been evaluated on their ability to diagnose mTBI, with modest success. The most widely studied marker in brain injury is Serum protein 100B (s100B), a low affinity calcium binding protein primarily expressed in glial cells and Schwann cells (Persson et al., 1987). s100B is a highly sensitive protein that can be found in both the CSF and blood within 6 h of mTBI (de Kruijk et al., 2001; Berger et al., 2002; Giacoppo et al., 2012). However, s100B has poor specificity, as it can be extracranially derived from various cell types elsewhere in the body (Bettermann and Slocomb, 2012). Furthermore, the function of this marker itself is not itself fully understood (Giacoppo et al., 2012), and thus should not be considered as a stand-alone marker for mTBI diagnosis. Similar conclusions have also been made about Neuron Specific Enolase (NSE), a neuronal damage marker. NSE was assumed to have high specificity to the brain, but was found be released into the serum as a result of hemolysis (Giacoppo et al., 2012; Papa, 2012; Žurek and Fedora, 2012), limiting its accuracy as a predictor of brain injury. Possibly more brain specific than NSE, however, is Ubiquitin Protein Hydrolase – 1 (UCH-L1), a marker of neuronal damage linked to TBI (Berger et al., 2012). UCH-L1 can be located in the serum of patients within 4 h of injury (Berger et al., 2012), but has yet to correlate with mTBI (Berger et al., 2012). Conversely, Myelin Basic Protein (MBP) measures axonal damage, and unlike s100B and NSE, has high brain specificity, but suffers from a delayed introduction into the blood stream (24–72 h post-injury) (Giacoppo et al., 2012).

Despite the limited success of various diagnostic mTBI biomarkers to date, current research provides reason for optimism. Recent work by Papa et al. (2012) has identified Glial Fibrillary Acidic Protein Breakdown Products (GFAP – BDP) elevated in the serum of mild and moderate TBI patients within a few hours of injury. Importantly, these increases were also correlated with GCS ratings, CT lesions, and neurosurgical interventions (Papa et al., 2012). Furthermore, GFAP itself appears to show high specificity to brain tissue, as multi-trauma does not affect its serum levels (Pelinka et al., 2005). Although caution must be taken in the interpretation of all correlative analyses, these findings are promising.

While markers such as GFAP and UCHL-1 measure astrocytic and neuronal damage, respectively, an important issue is raised regarding “proof of concept.” The pathophysiological mechanism(s) of trauma-induced injury in mTBI is unclear, and any single marker representing what may only be one “piece of the puzzle” has to be interpreted with caution. mTBI may encompass both neuronal and glial cell injury, with possible damage specific to axonal structures (Johnson et al., 2012) (see Table 1 for a summary of blood biomarkers in mTBI). In view of this, multiple markers used to assess the spectrum of brain tissue injuries that are mechanistically correlated with clinical symptoms would likely increase diagnostic accuracy. Furthermore, specific symptoms (e.g., loss of consciousness, amnesia) and types of trauma (e.g., focal versus, diffuse, direct head impact versus acceleration/deceleration injury not specific to the head) may need to be examined separately with greater scrutiny in order to create more direct connections between specific biological markers observed in the blood after injury and their precise underlying etiology.

**Table 1:** **Selected peripheral blood biomarkers of mTBI**.

Marker	Biological roles	Diagnostic	Prognostic	Injury mechanism	Reference
s100B	Calcium binding protein found in astrocytes and some neuronal cells	Lacks specificity, elevated levels found in the serum of multi-trauma patients	Poorly related to outcome as measured by return to work (RTW)	Suggests astrocyte damage/activation as a cellular sequelae to primary insult, as well as possible BBB disruption	Bazarian et al. (2006), Biberthaler et al. (2006), Naeimi et al. (2006), de Kruijk et al. (2001), Metting et al. (2012), Nygren-de Boussard et al. (2004)
	Found elevated in serum acutely post mTBI	Some validity for diagnosis of intracranial lesions (IL)	Even highly elevated levels have been shown full recovery	
NSE	Glycolytic enzyme, specific to the cytoplasm of neurons	Lacks sensitivity, and specificity; elevated levels found in blood resulting from hemolysis	Poor correlation between serum levels and GOS	Suggests acute neuronal damage	Meric et al. (2010), Naeimi et al. (2006), Berger et al. (2007), de Kruijk et al. (2001)
	Elevated post mTBI	
GFAP/GFAP BDP	Protein found in glial cells, major part of the astroglial skeleton	Promising, BDPs have high specificity and sensitivity	Poor predictor of RTW or GOSE	Suggests astrocyte damage, possible BBB disruption	Papa et al. (2012), Metting et al. (2012)
	Elevated within 1-h post mTBI	
MBP	One of two most abundant CNS proteins found in myelin	Detection of serum elevations may take up to 2–3 days, making it temporally unfavorable	Elevated serum levels may be related to poor outcome	Suggests structural axonal damage	Beers et al. (2007), Berger et al. (2005)
	Elevated in serum post mTBI	
Tau	Microtubule associated proteins located in CNS axons	Correlated with mTBI	Poor outcome predictor using 3-months PCS assessment as well as RPCQ	Suggests hyperphosphorylation resulting in formation of CNS tangles “tauopathy”	Guzel et al. (2010), Ma et al. (2008), Bazarian et al. (2006), Bulut et al. (2006), Small et al. (2013)
	Found elevated in the serum within 6 h of mTBI	Unable to identify patients with IL found on CT scans	
UCH-L1	Cytoplasmic protein found specifically in neurons	Not associated with pediatric mTBI	N/A	Suggests neuronal loss and disruption of the BBB	Berger et al. (2012)
SBDP145	One of the all-spectrin breakdown products, found in presynaptic terminals and axons	Not associated with pediatric mTBI	N/A	Suggests cell necrosis	Berger et al. (2012)

## Biomarkers of mTBI Prognosis

A prognostic biomarker is used to predict the clinical outcome of a disease or injury (Biomarkers Definitions Working Group, 2001; Petzold, 2007) and can be useful in guiding treatment strategies (Petzold, 2007). In mTBI, prognostic biomarkers generally have a twofold purpose: (1) to predict recovery; (2) to stratify risk for specific secondary pathological outcomes, such as PCS and chronic traumatic encephalopathy (CTE) (Bruns and Jagoda, 2009). However, prognostic markers may also have more precise predictive utility, such as aiding in the clinical decision to image patients (Biberthaler et al., 2006). Unfortunately, there are currently no accurate prognostic biomarkers of mTBI outcome, and more specifically, in risk stratification for the development of PCS and CTE. This problem is further compounded by the lack of diagnostic markers for CTE and PCS themselves (Lakhan and Kirchgessner, 2012).

The chronic affects of mTBI have received increasing media attention due to its impact on affected athletes and military personnel (Cancelliere et al., 2012). Among those who have had an mTBI, 50% will continue to experience cognitive, neurological, and behavioral symptoms such as headache, difficulty concentrating, anxiety, and depression (Begaz et al., 2006). This percentage drops to around 15% at the 1-year mark (Begaz et al., 2006), while some may continue to experience symptoms for years post-injury (NIH, 1999). This condition is known as PCS, and to date, little is known about its pathophysiological etiology (Nygren-de Boussard et al., 2004). Furthermore, chronic mTBI patients may also be at risk for CTE. CTE was originally identified over 80 years ago in “punch drunk” boxers, and presents as a neurodegenerative condition that worsens with age, quite often resulting in dementia, depression, memory loss, and even suicide. CTE may occur as a result of multiple brain injuries; 17% of those with repetitive head injuries go on to develop this syndrome (McKee et al., 2009).

To date, analysis of prognostic mTBI markers have been correlated to clinical decisions to image, as well as various clinical indices of recovery such as return to work (RTW) and the Glasgow Outcome Scale (GOS) (Bazarian et al., 2006; Beers et al., 2007; Metting et al., 2012). It was originally believed that s100B would be a useful clinical aid in predicting recovery and lowering the number of ill-advised CT scans (Ingebrigtsen and Romner, 1996). Unfortunately, recent assessments of this marker have revealed it is a poor predictor of intracranial risk (Morochovic et al., 2009), early neurological outcome (Piazza et al., 2007; Morochovic et al., 2009), and long-term post-concussion symptoms (Bazarian et al., 2006). In addition, increased levels of serum s100B have been noted in patients who have made a complete neurological recovery (Piazza et al., 2007). s100B has also been outperformed by GFAP in predicting long-term outcome (6 months), as reflected by the Glasgow Outcome Score Extended (GOSE) and RTW assessment (Metting et al., 2012). However, the authors of this study concluded that GFAP was still a weak overall predictor of outcome in mTBI (Metting et al., 2012). Also, NSE serum levels 1 month post mTBI are not correlated to outcome as reflected by the GOS (Meric et al., 2010), and the Tau protein has not been shown to correlate to PCS at 3 months (Ma et al., 2008) (see Table 1). Yet, the interpretation of these results must be approached cautiously, particularly in correlating mTBI recovery to assessments such as the GOSE and RTW. While possibly indicative of recovery, these correlates provide no objective and reliable pathophysiological determination. For example, it is quite possible that a patient suffering with PCS may RTW while not completely recovered, and conversely, some patients may have recovered well before they RTW. The GOSE and RTW assessments contain fairly ambiguous groupings such as “moderately disabled,” “good recovery,” and “previous work not resumed, but working on a lower level” (Metting et al., 2012). The utility of these types of scales, which are based on limited clinical symptom assessment, should be questioned, as the potential for subjective interpretation is high.

A greater number of longitudinal, multi-marker studies correlated with specific secondary symptoms (e.g., headaches, nausea, anxiety) may provide a useful kinetic background to identify candidate prognostic biomarkers. Beers et al. (2007), provide a useful framework through their study design, assessing multiple markers at multiple points in the acute period after trauma and at 6 months. Metting et al. (2012) also used a multi-marker approach, assessing GFAP and s100B as outcome markers in mTBI. More studies following this approach would be of use. Furthermore, diagnosing PCS and CTE still remain a challenge for clinicians, and until this is rectified, it will remain difficult to accurately predict patient outcome.

## Biomarkers of mTBI Pathophysiology

The fundamental pathophysiology of injury in mTBI is not understood, making it difficult to identify clinically functional biomarkers. Still, while many well-studied biomarkers have been criticized for their inability to suffice as stand-alone indicators of injury presence and outcome (Rothermundt et al., 2003; Giacoppo et al., 2012; Papa, 2012), investigating these markers has provided us with pivotal insight into the pathophysiology of mTBI. For example, clinically presented symptom clusters of mTBI are thought to be associated with neuronal (de Kruijk et al., 2001) and glial cell damage/activation (Pelinka et al., 2004; Metting et al., 2012), often specifically pertaining to axon structures (Bulut et al., 2006; Berger et al., 2007; Guzel et al., 2010), developing into what has become known as diffuse axonal injury (DAI) (Johnson et al., 2012). These developments, as well as our present understanding of secondary injury in mTBI are based on both experimental studies, and a large existing body of research on moderate and severe TBI. From this it has been suggested that secondary injury results from a maladaptive healing response that amplifies the damage incurred from primary injury. This is accomplished in a complex, multi-faceted nature, involving a variety of biochemical cascades related to a disruption in energy metabolism, protein synthesis and degradation, and dysfunction at the level of neural synapse (Greve and Zink, 2009; Cederberg and Siesjö, 2010; Jaerve and Müller, 2012; Johnson et al., 2012; Kan et al., 2012). However, studies on the biological sequelae resulting from the primary insult in mTBI are still relatively scarce.

Very few potential markers related to the advent of secondary injury have been assessed. Among these is SBD145, a cleavage product of αII-spectrin that is indicative of cell necrosis (Berger et al., 2012). However, in a single study assessing this marker in mTBI patients, no correlation between blood levels of SBD145 and predictive outcome in pediatric mTBI was found (Berger et al., 2012). The Tau protein has also been implicated in mTBI secondary injury, and has been found elevated in the serum of mTBI patients (Guzel et al., 2010). The term “tauopathy” has been associated with other neurological disorders such as Alzheimer’s and Parkinson’s disease, and is thought to be involved in the etiology of CTE (McKee et al., 2009). Tauopathy refers to the hyperphosphorylation of the Tau protein, causing biochemical alterations which lead to the formation of axonal tangles, ending in disruption of neuronal communication (McKee et al., 2009; Guzel et al., 2010). In view of this, Small et al. (2013) recently demonstrated increased Tau deposits in retired NFL players with histories of cognitive and mood symptoms. Although this preliminary study was constrained by a small sample size, it is the first report to date to identify such findings in live humans at risk for CTE. Further studies assessing this possible mechanism would help not only in CTE pathology, but would provide pivotal information about the degenerative processes occurring post mTBI.

There is strong evidence to support that TBI pathophysiology involves systemic innate and adaptive immune responses that are intricately involved in a communicative process between the periphery and brain parenchyma (Morganti-Kossmann et al., 2007; Cederberg and Siesjö, 2010; Giacoppo et al., 2012; Papa, 2012). In addition, recent experimental data in animals and humans in TBI have uncovered immunoexcitotoxicity as a novel pathological mechanism leading to CTE (Blaylock and Maroon, 2011). Thus, in parallel with the necessity of understanding the molecular pathophysiology of cell damage, there is a need for clinical studies in mTBI assessing markers of inflammation, in particular, those involving the recruitment of immune cells into both cellular and microvascular brain structures (Kochanek and Hallenbeck, 1992; Clark et al., 1994).

## Summary/Future Directions

Considering the health related and economic impact of mTBI (Dash et al., 2010; Giacoppo et al., 2012; Papa, 2012), an improved understanding of this condition is urgent. The prospect of using peripheral blood-based markers synergistically with current clinical diagnostic and prognostic assessments of mTBI is favorable for a variety of reasons: (1) it is more clinically accepted compared to other invasive procedures; (2) it is cost effective; (3) it may quickly and accurately provide specific information about the underlying pathophysiology of mTBI, which clinicians can then use in the diagnosis and formulation of treatment strategies.

Ultimately, the goal of biomarker research is to identify surrogate markers as an adjunct or replacement for specific clinical endpoints. By definition, the surrogate marker is one of achievement, as biomarkers are first considered “candidates” in hopes of acquiring the surrogate rank. However, in order to identify markers that may achieve this status, an in depth understanding of the cellular and molecular pathogenic mechanisms of mTBI is required. This point cannot be overlooked in any facet of biomarker research. Our current understanding of both primary and secondary mTBI pathology is poor, as is our understanding of PCS and CTE. Future research, whether experimental or clinical, would benefit by employing a more mechanistic based focus. Assessing a panel of markers will likely improve diagnostic and prognostic accuracy. Furthermore, in order to ensure proof of principle, specific mTBI symptoms need to be more directly linked to biological findings to establish causation. It will remain difficult to identify the mechanistic underpinnings of mTBI if biological findings are simply correlated to a cluster of symptoms. Controlling for specific symptoms/outcomes through greater cohort stratification may prove useful.

## Prospective Translational Techniques

High throughput “OMICs” technologies will continue to be invaluable moving forward with enhanced detection and characterization of novel blood-borne biomolecules. The field of proteomics shows great promise through its ability to characterize entire cellular environments (“Top-down approach”) and identify novel proteins involved in pathological processes (“Bottom-up approach”) (Colantonio and Chan, 2005). Wang et al. (2005) have published a very informative review on proteomic based research in TBI that captivates the essential importance and potential of this technology to the field.

Assessing the peripheral immune system in mTBI may potentially lead to invaluable information on the cause and/or consequence of secondary injury. Although severely understudied in mTBI, research in moderate and severe TBI has underlined neuroinflammation as an important process in secondary injury (Lenzlinger et al., 2001; Jaerve and Müller, 2012). In view of this, flow cytometry is a high throughput immunological technique with proven utility to elucidate pathological cell signaling pathways using human biological fluids, including whole blood, and is also used as a clinical diagnostic tool (Laerum and Farsund, 1981). Studies assessing the mechanisms underlying secondary injury in mTBI that incorporate conventional flow cytometry and imaging cytometry may yield important advancements to our understanding of both injury pathology and etiology of PCS and CTE.

In moving forward, it will be important for mTBI research to focus on elucidating pathophysiology. It would appear that a single marker will not achieve this, and to date, the search for biological indicators of mTBI has been met with limited success. However, there is much reason for optimism with regard to the ultimate potential of blood-based biomarkers. Recent data on such markers as GFAP-SBP and Tau have proved hopeful (Papa et al., 2012; Small et al., 2013). These markers may provide pivotal information into the underlying pathology behind mTBI and CTE respectively. Furthermore, advances in high throughput “OMICs” techniques such as mass spectrometry and imaging cytometry provide real potential in uncovering the biological mechanisms underlying mTBI and its chronic sequelae. These techniques will no doubt be intrinsically involved in the entire translational process of mTBI research, from the elucidation of pathophysiological mechanisms to the clinical implementation of validated diagnostic and prognostic biomarkers.

## Conflict of Interest Statement

The authors declare that the research was conducted in the absence of any commercial or financial relationships that could be construed as a potential conflict of interest.
